# Induction of pyroptosis in colon cancer cells by LXRβ

**DOI:** 10.4161/23723548.2014.970094

**Published:** 2015-02-24

**Authors:** Cédric Rébé, Valentin Derangère, François Ghiringhelli

**Affiliations:** 1 Institut National de la Santé et de la Recherche Médicale (INSERM); UMR 866; Dijon, France; 2 Centre Georges François Leclerc; Dijon, France; 3 Faculté de Médecine et de Pharmacie; Université de Bourgogne; Dijon, France

**Keywords:** Caspase-1, colon cancer, LXR, NLRP3 pannexin 1, pyroptosis

## Abstract

Liver X receptors (LXRs) have been proposed to have some anticancer properties. We recently identified a new non-genomic role of LXRβ in colon cancer cells. Under LXR agonist treatment, LXRβ induces pyroptosis of these cells *in vitro* and *in vivo*, raising the possibility of targeting this isoform in cancer treatment.

## Abbreviations

ABC: ATP-binding cassette transporter
ASC: apoptosis associated Speck-like protein containing a caspase activation recruitment domain
FAS: fatty acid synthase
LXR: liver X receptor
NLRP3: Nod-like-receptor pyrin domain containing 3
P2RX7, purinergic receptor 2X, ligand-gated ion channel: 7
ROS: reactive oxygen species
SREBF1: sterol regulatory element-binding transcription factor 1

## 

Liver X receptor α (LXRα or NRH1H3) and β (or NR1H2) belong to the nuclear receptor family. LXRα is expressed primarily in the liver, intestine, adipose tissue, and macrophages, whereas LXRβ is widely expressed in all tissues. After activation by natural ligands, such as oxysterols, these receptors increase the expression of target genes encoding proteins implicated in lipid metabolism, most particularly in cholesterol efflux (e.g., ATP-binding cassette transporter a1 [ABCA1] and ATP-binding cassette transporter g1 [ABCG1]) or fatty acid synthesis (e.g., fatty acid synthase [FAS] and sterol regulatory element-binding transcription factor 1 [SREBF1]).^1^

Previous studies have shown that LXRs are expressed in different cancer cell types (e.g., prostate, breast, ovarian, colon, glioblastoma, and melanoma) and are implicated in the control of cancer cell proliferation and cell death *in vitro* and *in vivo*.^2^

**Figure 1.**
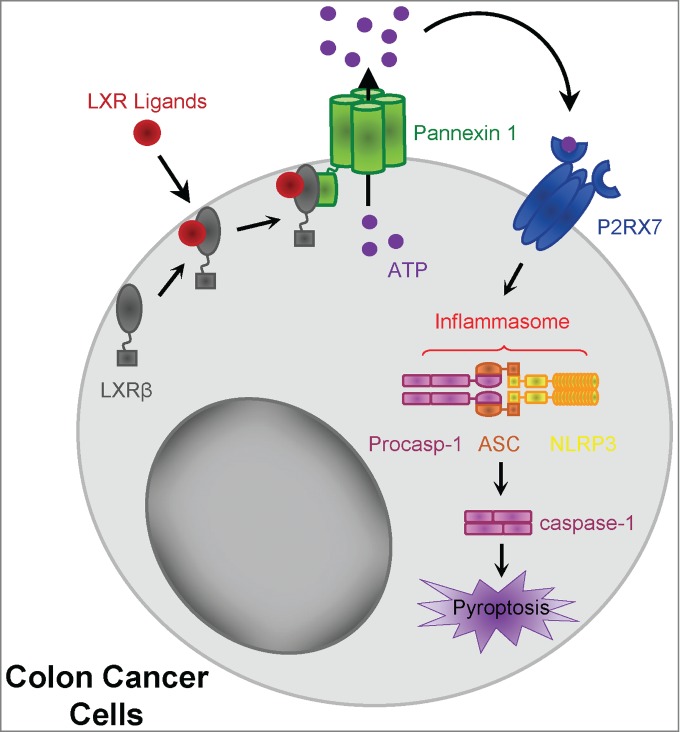
LXRβ-mediated pyroptosis in colon cancer cells. After treatment with LXR ligand, LXRβ (localized in the cytoplasm and at the plasma membrane) binds pannexin 1, leading to ATP release. Extracellular ATP activates the P2RX7 receptor, leading to assembly of the NLRP3 inflammasome with ASC and caspase-1 activation. Activated caspase-1 in turn induces colon cancer pyroptosis. P2RX7, purinergic receptor 2X, ligand-gated ion channel, 7; NLRP3, Nod-like-receptor pyrin domain containing 3; ASC, apoptosis associated Speck-like protein containing a caspase activation recruitment domain.

However, a common feature of these reports is that these mechanisms only involve the transcriptional activity of LXR, and more particularly of LXRα. We have recently identified a new non-transcriptional function of LXRβ, summarized in **Figure 1**.^3^ In our study, we report that LXR agonists can induce colon cancer cell death independent of any transcriptional activity. In particular, the first mechanistic events that induce cell death occur within the first minutes of treatment, whereas LXR target gene expression in these cells (i.e., *ABCA1* or *SREBF1*) was increased only after 24 hours. In addition, the transcription inhibitor actinomycin D had no effect on LXR-dependent cell death. Moreover, this non-genomic role of LXR ligands specifically depends on LXRβ (and not LXRα). These findings can be partly explained by the fact that LXRβ is not present in the nuclei of colon cancer cells, but instead exhibits a cytoplasmic, and to a lesser extent, plasma membrane localization. Within the first minutes of agonist treatment, LXRβ associates with pannexin 1, specifically with the intracellular C-terminal domain of this membrane channel, and induces ATP release.^3^ This domain has previously been described to be responsible for maintaining the closed conformation of pannexin 1, and cleavage of this region by caspase-3 leads to the opening of pannexin 1 and ATP release.^4^ Our results suggest that the opening of pannexin 1 might be induced not only by caspase-3 cleavage and removal of the C-terminal domain, but also by the binding of LXRβ to this domain.

Extracellular ATP released under LXR activation activates the purinergic receptor 2X, ligand-gated ion channel, 7 (P2RX7) pathway, leading to Nod-like-receptor pyrin domain containing 3 (NLRP3)/apoptosis associated Speck-like protein containing a caspase activation recruitment domain (ASC)-dependent caspase-1 activation.^3^ Caspase-1 is classically activated within macromolecular complexes called inflammasomes, and most often the well-characterized NLRP3 inflammasome.^5^ This complex primarily contains NLRP3, ASC, and caspase-1, which are all expressed in colon cancer cells.^3^ The NLRP3 inflammasome assembly has been shown to be mediated by reactive oxygen species (ROS) production, lysosome permeabilization (and cathepsin B activation), or activation of the ATP-mediated P2RX7 receptor pathway.^5^ In our study, only the P2RX7 pathway was required for caspase-1 activation by LXR agonist, independent of ROS production, lysosome acidification, or cathepsin B activation.^3^

Recently, it has been demonstrated that activation of caspase-1 not only leads to inflammation, but in certain circumstances causes an inflammatory form of cell death called pyroptosis.^6^ The features induced by LXR agonist are characteristic of pyroptosis: i.e., cell swelling to form a balloon-shaped vesicle around the nucleus, ATP release, caspase-1 activation (and to a lesser extent late caspase-7 activation), membrane permeabilization, and chromatin fragmentation. Moreover, we did not observe any activation of caspase-3, -8 or -9 in our setting.^3^ Pyroptosis was first described in myeloid cells infected by pathogens or bacteria, and to our knowledge this is the first demonstration that pyroptosis can be chemically induced in cancer cells without any bacterial or viral infection. The early membrane permeabilization leads to release of the cytoplasmic content into the extracellular environment, acting as a danger signal for neighboring immune cells.^7^ Among these signals, pyroptotic cells can release “find-me” signals, such as ATP, that will attract macrophages and facilitate the phagocytosis of dead cells and probably the priming of adaptive immune cells.^8^ Even though we have shown *in vivo* that an LXR agonist can reduce tumor growth by inducing LXRβ, pannexin 1, and NLRP3-dependent caspase-1 activation specifically in tumor cells, further investigations will be necessary to elucidate the precise interaction between pyroptosis of tumor cells and the immune system and to determine whether LXR agonists could induce immunogenic cell death. In addition to the direct cytotoxic effect of LXR agonist on tumor cells, LXR agonists might also mediate immune-dependent antitumor effects as LXR was described to directly regulate proliferation, differentiation, and activation of T helper Th1 or Th17 CD4 T cells, macrophages, or dendritic cells.^9^

Finally, Pencheva et al. recently published that LXRβ activation not only within the tumor cells, but also in stromal cells, induces the production of apolipoprotein E (ApoE) that suppresses melanoma invasion, angiogenesis, tumor progression, and metastasis.^10^ Taken together with these data, our findings raise the possibility of developing specific LXRβ ligands for cancer treatment.
